# Harnessing Biomarker Activatable Probes for Early Stratification and Timely Assessment of Therapeutic Efficacy in Cancer

**DOI:** 10.1002/EXP.20240037

**Published:** 2025-03-06

**Authors:** Qinrui Fu

**Affiliations:** ^1^ Key Laboratory of Maternal & Fetal Medicine of National Health Commission of China Shandong Provincial Maternal and Child Health Care Hospital Affiliated to Qingdao University Jinan China; ^2^ Institute for Translational Medicine College of Medicine Qingdao University Qingdao China

**Keywords:** activatable probe, biomarker, early stratification, molecular imaging, therapy effect, timely evaluation

## Abstract

The advancements in early stratification and timely evaluation of therapeutic effects have revolutionized the ability to assess curative outcomes promptly. The separation between diagnosis/treatment and timely assessment of treatment response, along with delays in evaluating therapy efficacy, are significant contributors to treatment failures. Traditional approaches for evaluating curative effects face challenges posed by tumor heterogeneity and resistance, making it challenging to determine the effectiveness of a given therapeutic regimen at an early stage in clinical practice. However, molecular imaging using activatable probes has overcome these obstacles and transformed the field by shifting focus towards developing functional probes for visualizing tumors as well as enabling early stratification or timely evaluation of therapy effects. In this article, we emphasize the importance of diverse activatable molecular imaging probes and provide insights into early stratification or timely evaluation of various therapies’ effects. Finally, we discuss the challenges faced in this field and propose future research directions.

## Graphical abstract

Considering the rapid progression of cancer in most cases, any delay in evaluating therapy outcomes poses a significant threat to patients’ lives. Consequently, it is imperative to implement stratification and conduct timely evaluations of therapeutic effects either before or during the early stages of cancer treatment. This article provides a comprehensive review of early stratification and timely evaluation of therapy effects through the utilization of activatable probes.

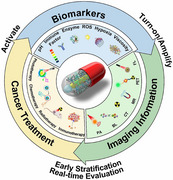

## 1 Introduction

Cancer is a major healthcare challenge that substantially increases the national health burden, causing millions of deaths every year [1]. Various treatment strategies have been developed, including chemotherapy [2, 3, 4], radiotherapy (RT) [5, 6, 7, 8], immunotherapy [9, 10], photothermal therapy (PTT) [11, 12], photodynamic therapy (PDT) [13, 14], among others [15, 16, 17, 18, 19]. However, due to tumor heterogeneity and resistance to therapies, patients show varying degrees of response to therapy effects across different tumor types and individuals [20, 21, 22, 23]. Therefore, early stratification and timely evaluation of therapy effect would be beneficial in triaging non‐responding patients away from toxic side effects of ineffective treatment. Importantly, this would also help gain valuable time for therapeutic regimen optimization, thereby achieving personalized precision treatment.

The current conventional stratification or evaluation approaches such as direct volumetric measurement method, histopathological and cytological assays usually take weeks after treatment [22, 24]. The clinical evaluation criteria for therapy effect mainly rely on indirect measurement of the tumor volume changes by using various imaging modalities including ultrasound imaging, X‐ray computed tomography (CT), bioluminescence, positron emission tomography (PET), and magnetic resonance (MR) imaging. However, these approaches often take several weeks or months after treatment to determine whether a given therapeutic regimen is effective [25, 26, 27, 28]. Considering the rapid progression of cancer in most cases, the delay in assessing treatment outcomes poses a significant threat to patients’ survival. Consequently, there is an urgent need for timely evaluation of therapeutic efficacy and stratification in the early stages of cancer management, and molecular imaging probes offer a breakthrough for this purpose.

Currently, molecular imaging probes have progressed through three stages of development. Initially, biomarker‐insensitive “always on” probes were used to detect concentration variances between normal tissue and the affected area. Nevertheless, these probes demonstrated limited signal specificity and sensitivity, resulting in a low signal‐to‐background ratio (SBR). The subsequent stage brought about activatable probes with biomarker‐responsive mechanisms, which aimed to improve imaging sensitivity and specificity. These probes remained dormant until triggered by specific biomarkers in the diseased microenvironment, offering superior SBR and a reduced limit of detection compared to the “always on” probes. Throughout these first two stages, conventional probes encountered a major limitation due to their reliance on absolute intensity‐dependent signal readings. This led to imprecise and misleading imaging outcomes, influenced by factors independent of the analyte causing fluctuations in absolute signal levels. In response to these challenges, ratiometric probes emerged as a promising solution. There are two widely recognized design strategies for developing ratiometric probes capable of imaging endogenous biomarkers. One strategy involves combining a biomarker‐insensitive reference signal with a biomarker‐responsive sensing signal within a single probe to eradicate errors linked to variations in probe concentration. The other method utilizes two reversible alterations in biomarker‐responsive signals to facilitate ratiometry measurements [29, 30, 31].

Over the past decade, significant advancements in molecular imaging utilizing activatable probes with biomarker responsiveness have revolutionized early stratification and timely evaluation strategies for assessing therapeutic efficacy [32, 33, 34, 35]. The activation of biomarkers associated with the tumor microenvironment precedes changes in tumor size, suggesting that these biomarkers could serve as “matchmakers” to establish a correlation between live imaging information and therapy effectiveness through responsive/targeting imaging techniques. This enables early stratification and timely evaluation of therapy response [36, 37]. To date, some biomarkers such as enzyme, immune response factors, inflammation factors activatable probes accompanied by molecular imaging such as CT, PET, MR imaging, fluorescence (FL) imaging, photoacoustic (PA) imaging, etc. have been extensively investigated for the purpose of stratifying, predicting, and evaluating therapeutic efficacy in the early stages of cancer treatment [38, 39, 40, 41, 42, 43].

**FIGURE 1:**
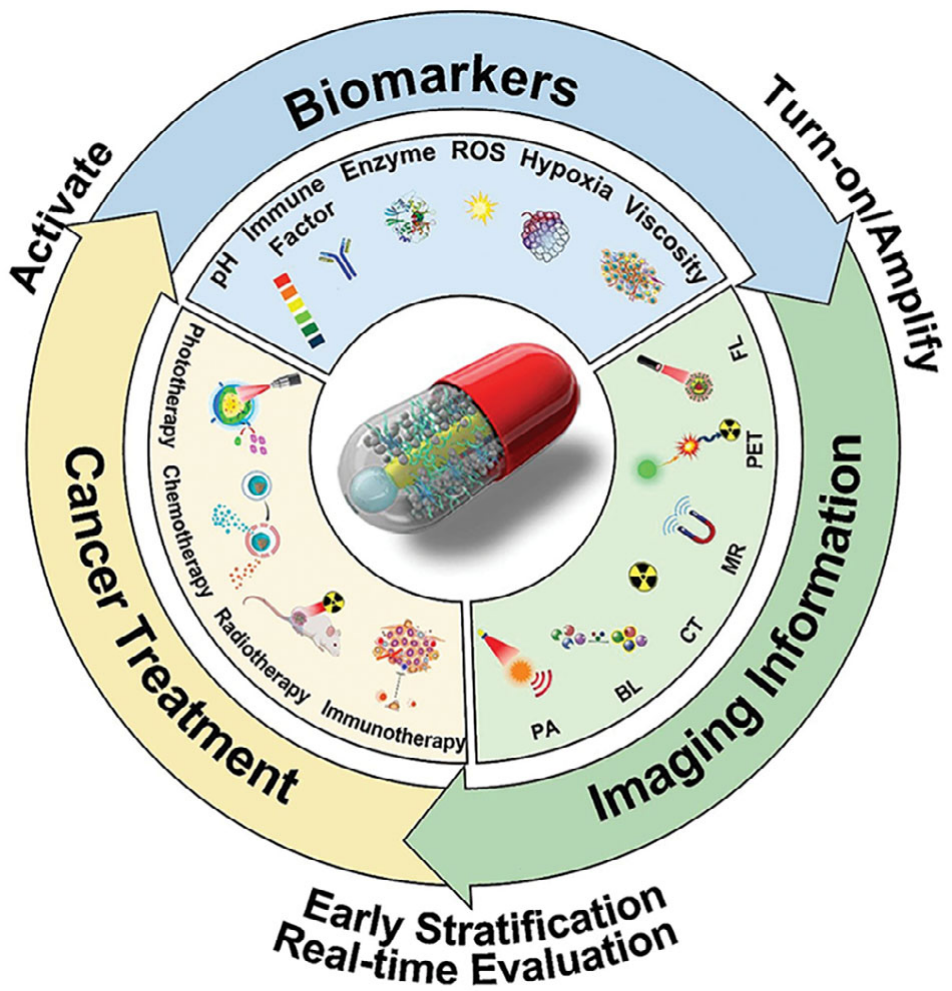
Schematic representation of early stratification and timely assessment of therapeutic efficacy using a biomarker‐responsive molecular imaging strategy.

As shown in the Figure 1, the biomarkers are secreted or activated after various treatment such as chemotherapy, RT, immunotherapy, PTT, and PDT. Diverse of activatable probes are designed based on the biomarkers’ properties, and combined molecular imaging in turn performs biomarkers‐responsive imaging of tumors. As a facilitator in connecting biomarkers, the relationship between imaging data and tumor suppression rate can be established, offering insights into early stratification and ongoing assessment of treatment efficacy. Despite promising prospects, scarcely any reviews thus for have comprehensively summarized applications of activatable molecular imaging probes in the early stratification and timely evaluation of therapy effect. This review provides a comprehensive overview of notable advancements in the stratification and timely assessment of therapy effectiveness using activatable molecular imaging probes that have been reported in recent years. Additionally, potential prospects for innovating activatable probes to enable early stratification and timely evaluation of therapy effectiveness are also discussed.

## 2 Early Stratification of Therapeutic Efficacy Using CT Imaging Strategy

CT imaging is one of the most practical diagnostic tools in hospitals today because of its advantages in availability, efficiency, and cost [44, 45]. The principle of CT imaging is as follows: As an X‐ray beam passes through a patient's body, it undergoes attenuation due to absorption or deflection by body‐tissue or contrast agents. The detectors, situated on opposing sides of the patient, capture the attenuated X‐rays and subsequently convert them into digital data for image acquisition [46]. CT imaging is often used to evaluate therapy efficacy by in vivo detecting the macroscopic varieties in tumor volume [47], however, in the early phase of cancer treatment, apoptosis and necrosis of tumor cells often precede changes in tumor volume, thus resulting in lagging evaluation and hindering the optimization of therapeutic regimen and causing the adverse progression of the diseases. Therefore, developing evaluation techniques to predict early on whether a given therapeutic regimen will produce the desired therapy effect in an individual patient is of great significance, particularly for patients with advanced cancer where therapeutic options become increasingly limited as the patient's condition deteriorates. Since the complexity and heterogeneity of the tumor, and the diversity of patient responses to treatment, a given therapeutic regimen does not work for all patients. For example, the success rate of immunotherapy is only at most 30%. Furthermore, the efficacy of immunotherapy can only be assessed at 2 months after the initiation of treatment through traditional curative effect evaluation methods. Therefore, early stratification of diverse patient populations or timely assessment of therapeutic efficacy is imperative for enhancing treatment decision‐making.

**FIGURE 2:**
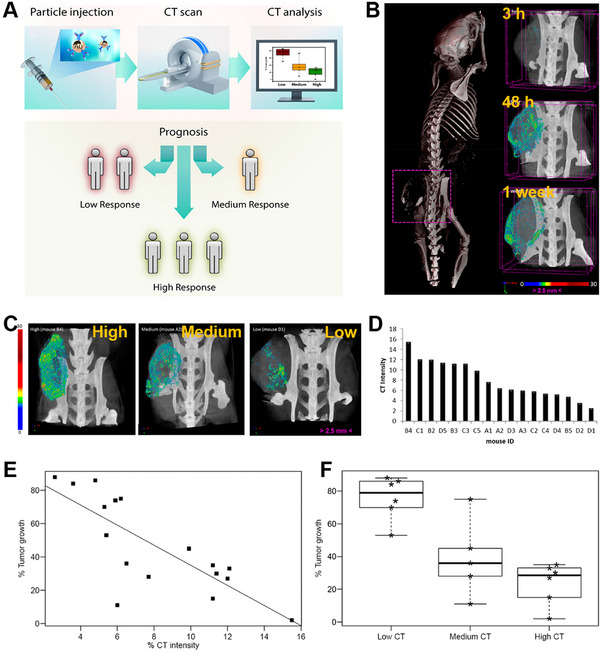
(A) Early prediction of immune response via CT imaging nanoprobe strategy. (B) CT images exhibited αPDL1‐AuNPs accumulation at the tumor. (C) CT scans depicting mice exhibiting signals of varying intensities: low, moderate, or high. (D) Variations in the accumulation of αPDL1‐AuNP nanoprobe observed among a group of seventeen mice. (E) Examined the relationship between CT signal intensity and tumor progression through correlation analysis. (F) Mice were assigned to 3 groups (low, medium, and high) based on the signal intensity. Reproduced with permission from ref. [48]. Copyright 2017, American Chemical Society.

In response to this need, Popovtzer et al. developed a programmed death ligand 1 antibody (*α*PDL1) modified gold (Au) nanoparticles (NPs) probe (termed *α*PDL1‐AuNPs) that integrated both diagnostic and therapeutic functions into a single NPs formulation for CT imaging‐mediated early stratification and early prediction of therapeutic response to immunotherapy (Figure 2A) [48]. The in vitro experiment was conducted to assess the effectiveness of *α*PDL1‐AuNPs as inhibitors of immune checkpoints. The application of *α*PDL1‐AuNPs resulted in a significant reduction in PDL1 expression within MC38 colon carcinoma cells, indicating that the ligand responsible for immune checkpoint activation on the cell surface could interact with αPDL1‐AuNPs and promote T cell activation. In addition, in vivo experiments indicated *α*PDL1‐AuNPs could accumulate and penetrate to the periphery and intratumoral tissue thereby enhancing tumor uptake of the nanoprobe, the nanoprobe reached its highest accumulation level 48 h after injection (Figure 2B). CT imaging conducted 48 h after injection exhibited significant variation in the uptake of αPDL1‐AuNPs among multiple tumor‐bearing mice, highlighting the diverse responses of tumors to immunotherapy owing to their intricate and heterogeneous nature (Figure 2C,D). Subsequently, an early assessment of the therapeutic response was conducted. A group of twenty mice with tumors were administered *α*PDL1‐AuNPs intravenously. The mice then underwent a CT scan after 48 h and tumor size measurement after 8 days following the injection. The findings revealed significant variations in the mice's individual responses to the therapy's effectiveness. A strong linear relationship (*R*_2_ = 0.6162) was found between the tumor volume at day 8 and the quantitative CT signal at 48 h after injection (Figure 2E), indicating that it is possible to predict therapeutic response as early as 48 h following treatment. the percentage of tumor growth in mice with low and high CT signals showed a minimal standard error. Furthermore, there was a strong correlation between the CT signal and T cell infiltration, indicating that the combination of CT imaging and αPDL1‐AuNPs nanoprobe could be utilized for early stratification and accurate prediction of therapeutic response (Figure 2F). This technology may provide new perspectives for noninvasive early stratification as responders or non‐responders of patients.

## 3 Early Stratification or Timely Assessment of Therapeutic Efficacy Using PET Imaging Strategy

PET could offer in vivo quantitative information with ultrahigh specificity, sensitivity, and excellent safety [49, 50, 51]. The principle of PET is as follows: The probes utilized in PET are radiolabeled with radionuclides that undergo decay through the emission of a positron. Upon release from the nucleus, the positron travels a short distance within the surrounding tissue before it annihilates by combining with an electron. This travel distance is commonly referred to as the positron range. Subsequent to annihilation, the combined mass of the positron and electron converts into energy, resulting in simultaneous emission of two 511 keV γ‐rays at approximately 180° angles to each other. These paired γ‐rays are detected by surrounding detectors, enabling recording of information pertaining to positron annihilation events. By acquiring a substantial number of coincidence events, data can be obtained for image reconstruction that provides spatial distribution details regarding radioactivity over time [52]. Developing appropriate PET probes is the central to PET imaging. In recent years, biologically interesting PET imaging nanoprobes have been developed for assisting drug development and clinical diagnosis [53, 54]. However, reports of using PET techniques to early predict and timely assess therapy effect are still rare.

In 2018, Gambhir et al. developed a ^64^Cu‐labeled antiOX40 antibodies PET probe (termed ^64^Cu‐DOTA‐AbOX40) for assessing the changes of cytotoxic T lymphocytes in clinically relevant cancer vaccine applications [55]. The OX40‐targeted ^64^Cu‐DOTA‐AbOX40 probe could more accurately indicate an effective immune response due to the OX40 is a cell‐surface biomarker of T cell activation. In vivo experiment showed a strong negative correlation between tumor response and PET signal intensity in tumors on day 2‐post treatment, thus the PET could be used to early predict immunotherapy effect.

**FIGURE 3:**
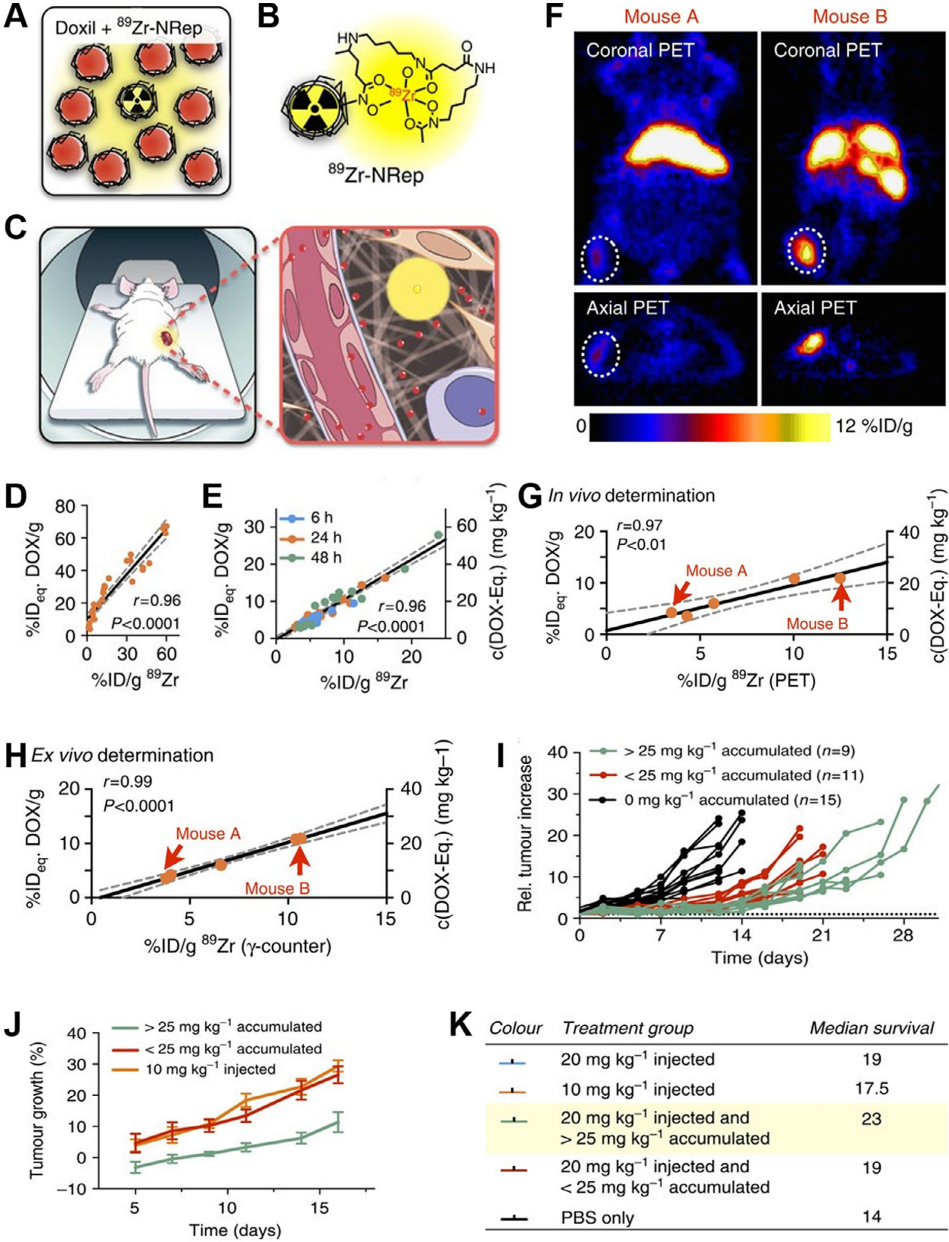
(A) ^89^Zr‐NRep doped Doxil formulation. (B) Schematic illustration of ^89^Zr‐NRep. (C) Schematic illustration of noninvasive quantification of DOX delivery through co‐injecting ^89^Zr‐NRep and Doxil. (D) Association between ^89^Zr‐NRep and DOX levels in the bloodstream. (E) Correlation between ^89^Zr‐NRep and DOX uptake. (F) PET images of different treatment using ^89^Zr‐NRep. (G) Correlation of ^89^Zr‐NRep uptake values derived from PET imaging and DOX tumor concentrations. (H) Correlation of DOX tumor concentrations and tumor relevant activity. (I) Individual tumor growth rates. (J) Average tumor growth rates. (K) Median survival of mice. Reproduced with permission from ref. [56]. Copyright 2016, Springer Nature.

The effectiveness of chemotherapy can be assessed by utilizing PET imaging in conjunction with probes. For example, Reiner et al. designed a nanoreporter ^89^Zr‐NRep and nanoliposomal doxorubicin (Doxil) consisting of an ^89^Zr labeled pegylated liposome via a desferrioxamine B modified phospholipid (Figure 3A,B) for early prediction of therapeutic outcome [56]. Co‐ injecting ^89^Zr‐NRep and Doxil (Figure 3C), strong association between ^89^Zr‐NRep and DOX levels in the bloodstream (Figure 3D), as well as strong correlation between ^89^Zr‐NRep and DOX uptake in tumor (Figure 3E) were observed. Importantly, the slope of this correlation was close to one, which indicated that %ID per g of ^89^Zr was roughly equivalent to %ID per g DOX, thus suggesting that the ratio of Doxil to ^89^Zr‐NRep remained constant after tumor accumulation when nanoreporter was injected. In addition, in vivo PET imaging experiment showed ^89^Zr‐NRep PET enabled accurate quantitation of DOX in tumor accumulation (Figure 3F–H). Therefore, ^89^Zr‐NRep PET could be used to early predict therapeutic outcome. The mice were divided into 3 groups (controls, >25 mg kg^−1^ DOX or <25 mg kg^−1^ DOX) based on PET signal intensities. At day 7, the tumor volume in >25 mg kg^−1^ DOX group was significantly smaller than that in <25 mg kg^−1^ DOX group (Figure 3I), moreover, median survival for >25 mg kg^−1^ DOX group was greater than that for <25 mg kg^−1^ DOX group and the control group (Figure 3J,K). The results illustrated therapy effect could be predict at the early stage through ^89^Zr‐NRep PET imaging.

The molecular imaging probes exhibit an inactive state in non‐target tissues initially, but upon reaching the disease target tissue, they are triggered by the biomarker present within tumor microenvironments to enhance or intensify the signal. This enhances both the specificity and sensitivity of disease detection and evaluation [57]. An important means by which cancer cells are eliminated during the immune response is through the release of granzyme B, a serine‐protease that is produced by natural killer cells and CD8^+^ T cells. Mahmood et al. designed a ^68^Ga labeled peptides of targeting granzyme B (termed ^68^GaNOTA‐GZP) that selectively and quantitatively interact with granzyme B for distinguishing immune responses from non‐responders and therapeutic assessment in early stage through PET imaging, thus achieving early stratification of therapy response [58].

## 4 Early Stratification of Therapeutic Efficacy Using MR Imaging Strategy

The generation of tissue images in MR imaging is achieved through the measurement of the interaction between an external magnetic field and the magnetic moment of water protons [59]. MR imaging, providing excellent anatomical accuracy in soft tissues and detailed information about macroscopic structure, has long been recognized as one of the well‐established and most important tools in medical diagnosis and research [60, 61, 62, 63, 64]. However, traditional MR imaging does not meet the requirements for high sensitivity and there is currently no effective method for stratifying therapeutic response and predicting therapy effect at an early stage. Hence, it is highly desirable to devise a timely evaluation system that enables early prediction and assessment of therapy effect using MR imaging.

It is widely believed that the primary driving force behind the accumulation of nanoparticles in tumor tissues is due to the “enhanced permeability and retention” (EPR) effect. However, how to predict the degree of enrichment and clinical efficacy of therapeutic NPs remains a challenge. To this end, Weissleder et al. designed a ferumoxytol NPs (FMX) with size of 30 nm for predicting accumulation and therapeutic effect of NPs via MR imaging in vivo. After intravenous injection of FMX, the correlation between imaging signal and tumor volume was constructed for quantifier of EPR and patient stratification [65]. Immunotherapy has established a novel paradigm for the control and therapy of diseases, resulting in considerable breakthroughs in the clinic. All the same, Immunotherapy continues to encounter a significant obstacle in achieving high patient response rates, primarily attributed to unavoidable factors including tumor heterogeneity, immunosuppression, variations in cancer types and stages of progression, as well as the development of adaptive resistance. Early identification of potential responder patients and non‐responder patients to immunotherapy is significance for optimizing treatment decisions. Mitchell et al. developed an RNA‐modified magnetic liposomes (IO‐RNA‐NPs) that delivered RNA to dendritic cells (DCs) and activated DCs to turn on antitumor immunity and early predict antitumor response with MR imaging. The results in the tumor model showed tumors size on 2−5 weeks post‐treatment of “responder” predicted with MR imaging information at 48 h after vaccination was obviously smaller than that of MR imaging‐predicted “non‐responders” [66].

**FIGURE 4:**
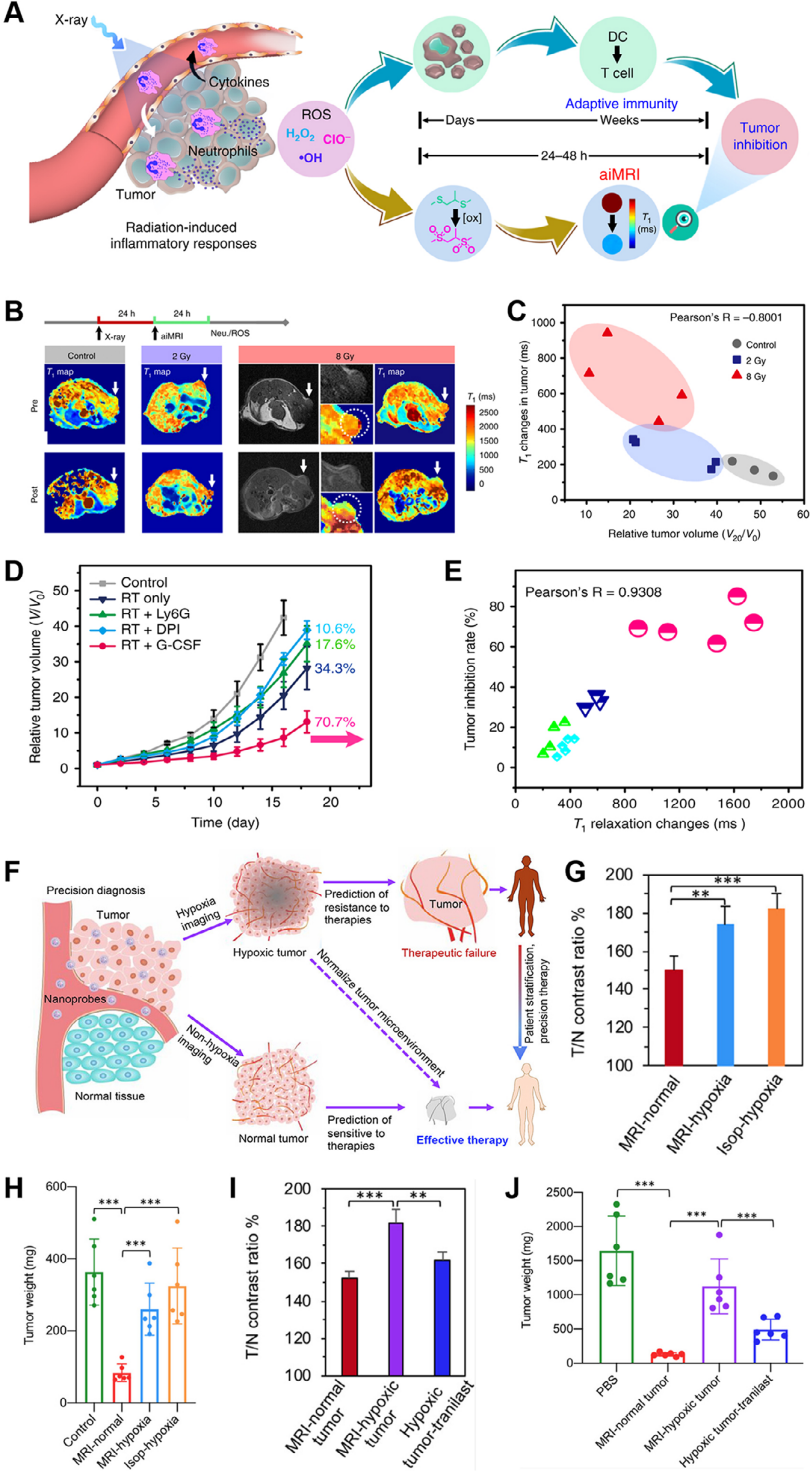
(A) The inflammation magnetic resonance imaging (aiMRI) enabled early stratification of radiotherapy (RT) response. (B) The *T*_1_ phantom images of tumors before and after contrast administration, as well as (C) the correlation between variations in *T*_1_ relaxation time at 1–2 days and the relative tumor size at 20 days after RT in U87MG tumor models. (D) The tumor volume ratio in mice with 4T1‐induced tumors. (E) Correlations between *T*_1_ relaxation time measurements and the level of tumor suppression. (F) Development of MR‐CA nanoprobes with activatable properties for imaging hypoxia and early assessment of treatment outcomes. (G) Stratification of tumors according to the T/N contrast ratios, and (H) tumor weight of different hypoxia condition after RT. (I) Stratification of tumors based on the T/N contrast ratios, and (J) tumor weight of different hypoxia condition after immunotherapy. (A–E) Reproduced with permission from ref. [67]. Copyright 2020, Springer Nature. (F–J) Reproduced with permission from ref. [68]. Copyright 2021, American Chemical Society.

Tumor heterogeneous and radioresistance are two main causes for unpredictable therapy effect. Stratification of therapeutic responses and prediction of therapy effect in the early stage may be greatly beneficial for better cancer control and therapeutic regimen optimization. Chen et al. reported a reactive oxygen species (ROS)‐responsive hybrid nanovesicle (IO‐Gd NVs) to stratify RT response and early predict RT effect through activatable inflammation MR imaging (aiMRI) method (Figure 4A) [67]. At 24 h after RT with different irradiation doses, the aiMRI was performed to quantify *T*_1_ relaxation time changes in tumors of U87MG mouse models (Figure 4B). The relative volume of the tumor was measured 20 days after irradiation to monitor its size. A significant inverse relationship (Pearson's correlation coefficient, *R* = −0.8001) was observed between the changes in *T*_1_ relaxation time at 24 h post‐radiotherapy and the relative tumor volume at day 20 (Figure 4C), which suggested the great promise for stratifying the RT outcomes using aiMRI. Following that, the *R* = 0.9308 were acquired from the correlations between the tumor inhibition ratios at 18 days and the *T*_1_ relaxation time changes at 1–2 days after various treatments in Balb/c mouse 4T1 tumor models (Figure 4D,E), further indicating the quantitative aiMRI method enabled stratification of RT effect regardless the mouse models.

Activatable nanoprobes in combination with MR imaging can also be used to predict responses to both RT and immunotherapy. For example, Mi et al. developed a MR contrast amplification (MR‐CA) nanoprobes consisting of PEGylated polyanions and Mn^2+^‐doped CaP NPs for quantitative hypoxia imaging to predict both RT and immunotherapy effect, and normalization of the hypoxic tumor microenvironments to improve therapeutic outcomes (Figure 4F) [68]. Firstly, BxPC3 tumor‐bearing mice was established for predicting responses to RT. Tumors were stratified based on tumor‐to normal tissue (T/N) contrast ratio, mice were divided into normal group (<160) and hypoxia groups (>160) using a T/N value of 160 as the dividing line (Figure 4G). Following a period of 26 days post X‐ray irradiation, the tumor weight exhibited a significant reduction in the normal group compared to the hypoxia group (Figure 4H). The result indicated that MR‐CA imaging of hypoxia could successfully achieve tumor stratification for predicting RT effect. Different approaches but equally satisfactory results, early prediction of responses to immunotherapy was also achieved by stratifying tumors based on T/N value in C57BL/6 tumor‐bearing mice. Interesting, tranilast enabled normalization of the hypoxic tumor microenvironments for lowering T/N value and sensitizing treatments (Figure 4I). After 18 days of immunotherapy, the tumor weight in the hypoxia group showed a significant increase compared to both the tranilast‐treated hypoxia group and normal group (Figure 4J).

In addition to RT and immunotherapy effect could be predicted, the inflammatory therapy effect could also be predicted in early stage via timely MR imaging approach. For instance, Deng et al. devised nanoprobes that respond to ROS and can be used for theranostics of inflammation associated with ROS as well as early assessment of treatment efficacy through MR imaging [69]. The strong correlation observed between the changes in relaxation (Δ*R*_1_) and the concentration of H_2_O_2_ may indicate the level of inflammation and enable early prediction of the effectiveness of anti‐inflammatory therapy.

## 5 Timely Assessment of Therapeutic Efficacy Using FL Imaging Strategy

**FIGURE 5:**
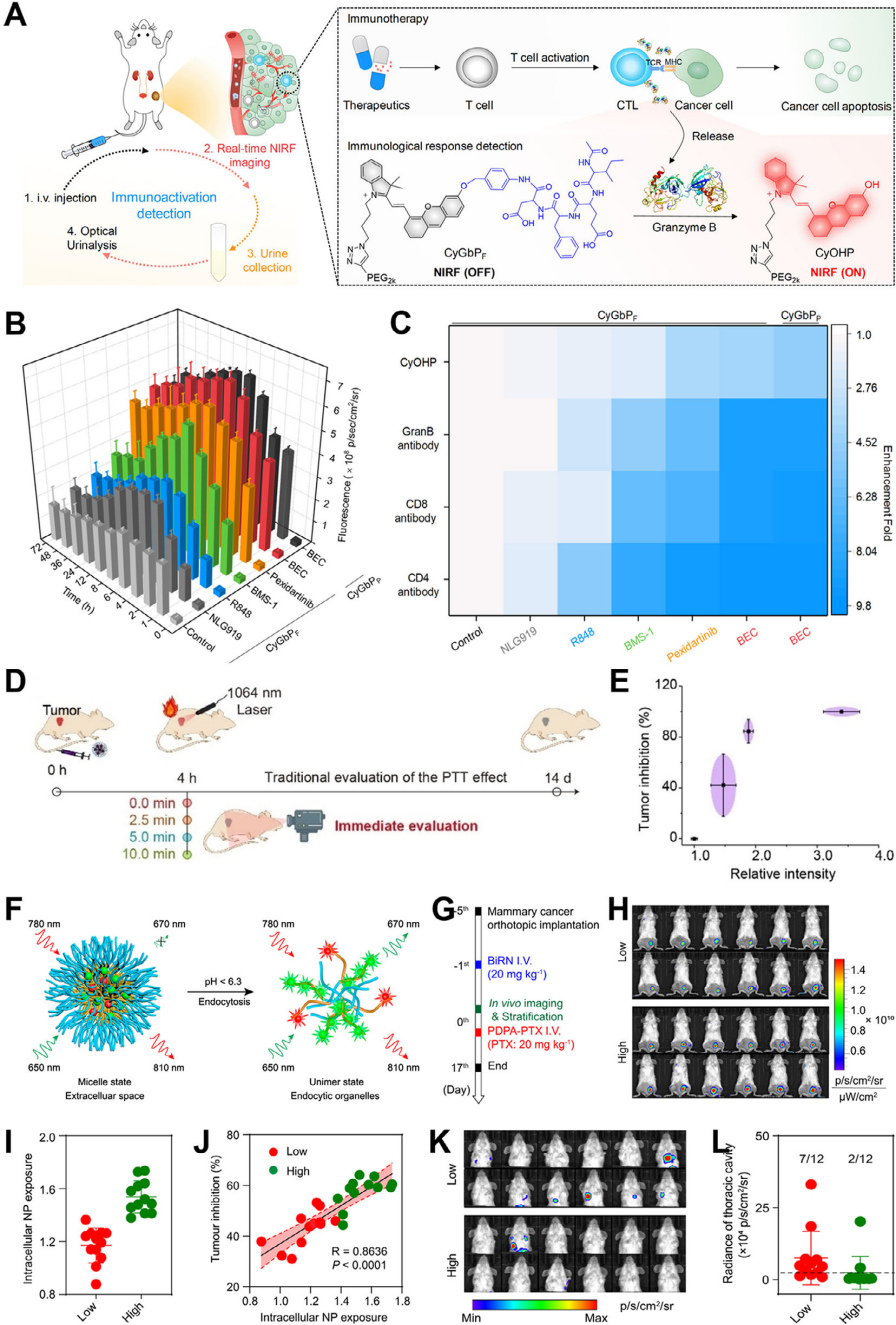
(A) The FL imaging mechanism of CyGbPF probe. (B) Measurement of the FL signal in the tumor region at various time points after injecting CyGbPF or CyGbPP. (C) Heat map of FL signals and biomarkers in the tumor tissues. (D) Schematic illustration of FL imaging based timely evaluation and conventional tumor volume changing assessment of PTT effect. (E) Correlation between relative FL intensity and tumor inhibition rate. (F) Schematic illustration of the ratiometric BiRN nanoprobe. (G) Experimental design for predicting chemotherapy response. (H) FL tumor images acquired 24 h after the administration of BiRN. (I) Stratification of tumors based on intracellular NPs level. (J) Correlation between intracellular NPs level and tumor inhibition rate. (K) Lung metastases images. (L) Quantitative analysis of lung metastases signal in mice. (A–C) Reproduced with permission from ref. [79]. Copyright 2020, American Chemical Society. (D, E) Reproduced with permission from ref. [80]. Copyright 2022, Wiley‐VCH. (F–L) Reproduced with permission from ref. [81]. Copyright 2021, Springer Nature.

FL imaging is a highly sought‐after molecular imaging technique that plays a crucial role in enhancing the accuracy of diagnosis, treatment, and surgical procedures facilitated by imaging in both basic research and clinical settings [70, 71, 72, 73, 74]. Compared with the conventional molecular imaging modalities such as CT, PET, and MR imaging, FL imaging has the merits of excellent spatiotemporal resolution and low cost [75, 76]. Most of the FL imaging reported so far are focused on diagnosis and treatment of diseases, however, no direct contact and timely information is provided for the therapy effect, which requires additional methods to evaluate. The timely anticipation and ongoing assessment of treatment efficacy play a crucial role in clinical settings as they help avoid prolonged periods of ineffective therapy [77, 78]. Pu et al. designed two immunoactivation‐related biomarkers (granzyme B) responsive near‐infrared macromolecular nanoprobes (CyGbPF and CyGbPP) for timely evaluation of immunotherapy effect (Figure 5A) [79]. In the presence of granzyme B, FL_707nm_ signal of both CyGbPF and CyGbPP could be specifically turned on and amplified more than 20‐fold, as well as activated immune cells (CTLs) could be effectively distinguished from other cells in vitro. In addition, the CyGbPF and CyGbPP could be used to accurately detect granzyme B in the tumors of various immunotherapeutic‐treated living mice, a desirable correlation between FL signal and immunotherapy‐based biomarkers such as CD^8+^ and CD^4+^ secreted by the T cells was observed (Figure 5B,C), thus this activatable FL imaging strategy could be employed to timely evaluate the immunotherapy effect.

Receiving a timely response to immunotherapy and monitoring the activity of granzyme B in tumor cells are crucial aspects. Hence, it is essential to efficiently capture the activity by simultaneously administering probes that respond to granzyme B and immunotherapy agents to the same cells. To this end, Kulkarni et al. developed a granzyme B activatable nanoprobes consisting of a granzyme B responsive peptide substrate bridged a fluorescence resonance energy transfer (FRET) pair and immunotherapy drug. The utilization of nanoprobes enables the concurrent administration of an immune checkpoint inhibitor (*α*PDL1) and imaging probe to the tumor area, facilitating timely monitoring of time‐sensitive granzyme B activity as a direct method for early assessment of immunotherapy efficacy [82]. The FL imaging signal intensities exhibited a strong association with the expression level of granzyme B, infiltration of T cells, and death of tumor cells. This highlights the remarkable precision in identifying responders using activatable nanoprobes treatment, even when there were no significant variations observed in tumor volume. In summary, the utilization of granzyme B activatable nanoprobes and FL imaging strategy enables the attainment of early stratification and timely assessment of immunotherapy efficacy in both in vitro and in vivo experiments.

The FL imaging strategy can be used not only for timely evaluation of immunotherapy effect, but also for evaluating photothermal therapy (PTT) effect in real time [83]. For example, Lin et al. developed LET‐1052, an organic dye that can be activated in acidic tumor microenvironments (TME) to enable second near‐infrared (NIR‐II) PTT. Subsequently, they evaluated the therapeutic efficacy based on changes in viscosity [80]. After the LET‐1052 probe was administered intravenously, its absorption intensity at 1052 nm exhibited a 3.4‐fold increase due to the protonation of nitrogen atoms by the acidic TME. This led to electron rearrangement and the formation of an extended polymethine π‐conjugation system, enabling effective NIR‐II PTT when exposed to a 1064 nm laser. After a 10‐min session of PTT, the initial fluorescence intensity in the tumor area, as detected by near‐infrared (NIR‐I) light, exhibited an immediate 3.5‐fold increase. This enhancement can be attributed to the PTT‐induced demise of tumor cells, which subsequently elevated intracellular viscosity and hindered intramolecular rotation. Consequently, the pH/viscosity activatable probe hold promise for timely assessment of PTT efficacy. Utilizing various durations of NIR‐II laser exposure, a direct relationship between the intensity of NIR‐I fluorescence in the tumor region and the rate of inhibition in tumor growth (Figure 5D,E) was observed. This suggested that an activatable fluorescence imaging approach enabled early prediction and timely assessment of PTT effectiveness.

One of the primary difficulties faced by conventional FL probes is their reliance on absolute intensity‐dependent signal readout, leading to imprecise imaging and detection outcomes due to numerous analyte‐independent factors causing fluctuations in the absolute signal. On the other hand, ratiometric FL probes offer an inherent self‐calibration mechanism for correcting signal intensity, thereby enabling more dependable and sensitive sensing [84]. Wang et al. developed a binary ratiometric nanoprobe (named as BiRN) consisting of FRET donor (PDPA‐Cy5) and FRET acceptor (PDPA‐Cy7.5) that could unambiguously distinguish NPs at intracellular and extracellular locations, allowing quantitative imaging of NPs internalization in vivo [81]. After endocytosis, when there is a sudden decrease in pH within the early endosome, the BiRN nanoprobe was disassembled and FRET effect disappeared, thereby forming FL_670_/FL_810_ signal (Figure 5F) for ratiometric quantitation of NPs internalization. The ratiometric FL imaging results showed only about twenty percent of the NPs in the tumors were internalized into the cancer cell components. Following that, the chemotherapy (PDPA‐PTX) response in 4T1 orthotopic tumor model was predicted using BiRN FL nanoprobe (Figure 5G). The mice were stratified into low‐ and high‐endocytosis groups based on the level of intracellular NPs calculated by ratiometric FL intensities (Figure 5H,I). After the administration of PDPA‐PTX NPs through intravenous route, a strong correlation (*R* = 0.8636, *P* < 0.001) was observed between the level of NPs inside cells and the rate of tumor inhibition on day 16 after chemotherapy (Figure 5J). Additionally, there was a higher percentage (58.3%) of mice with lung metastases in the low group compared to that (16.7%) in the high group (Figure 5K,L). These results demonstrated that this ratiometric FL imaging nanotechnology provided a valuable tool to early evaluation of chemotherapy effect and early stratification of patients for personalized cancer therapy.

## 6 Early Assessment of Therapeutic Efficacy Using Afterglow Luminescent Strategy

Afterglow imaging relies on chemical or energy traps to capture photons during pre‐irradiation and gradually release them after laser irradiation stops [85, 86, 87, 88]. High SBR imaging and precise detection of specific analytes in living tissues are two matters of great concern to basic biomedical research and clinical practice. Compared with FL imaging that suffers from unavoidable photobleaching and inevitable auto‐fluorescence of tissue, afterglow luminescent can circumvent the disadvantages of FL imaging and provide higher sensitivity and SBR, and deeper imaging depth because it is an unexcited imaging technique that retains absorbed light energy and subsequently releases photons at a gradual pace [89, 90, 91, 92]. Even so, there are few reports that early evaluation of therapy effect using afterglow imaging technique.

**FIGURE 6:**
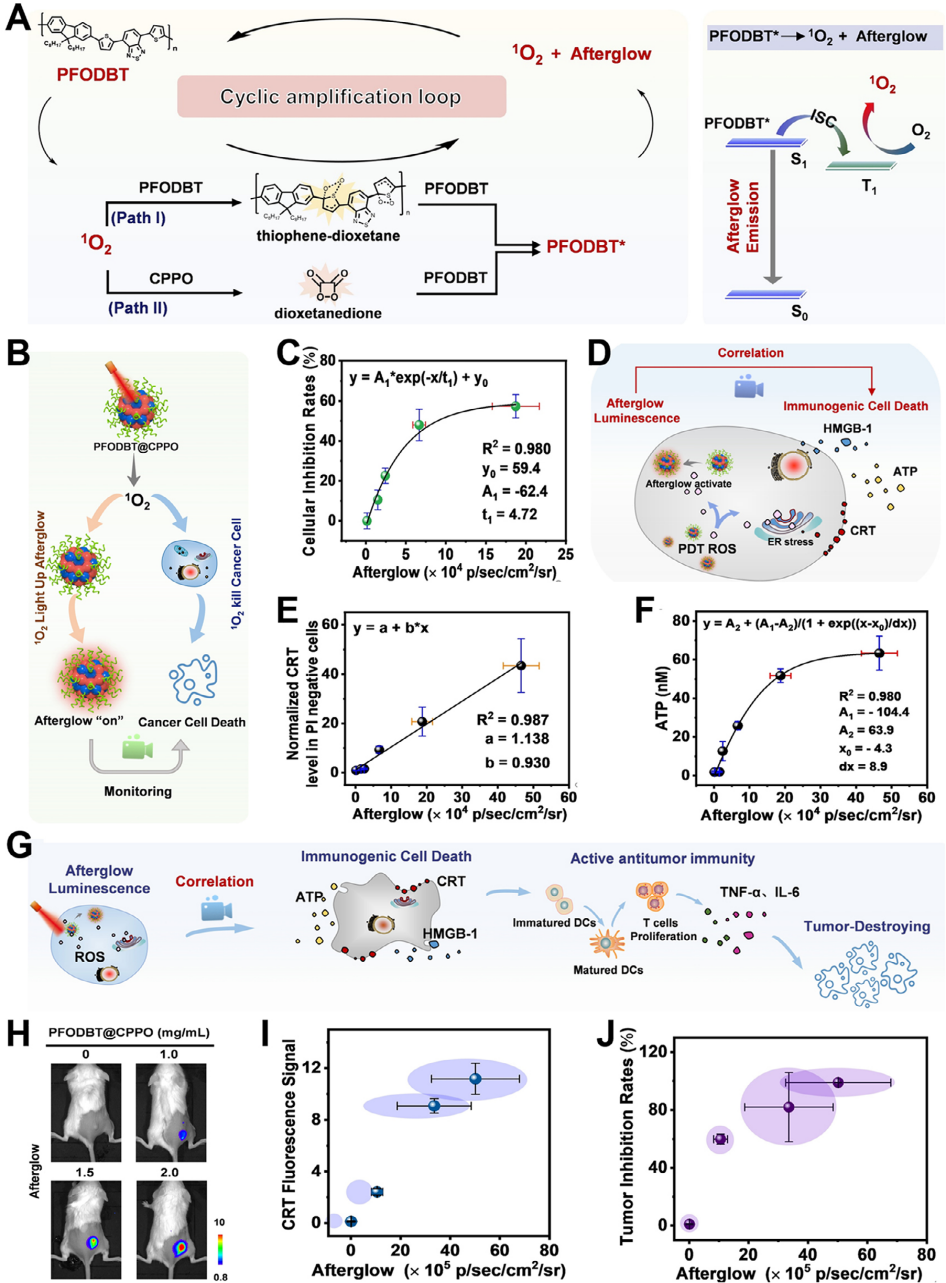
(A) Schematic depiction of the mechanism underlying afterglow luminescence. (B) The cellular inhibition rate was correlated with the monitoring of ^1^O_2_‐induced afterglow luminescence. (C) Relationship between the impact of afterglow and PDT efficacy at the cellular level. (D) Illustration in diagram form depicting the correlation between signal intensities of afterglow and levels of expression for immunogenic cell death (ICD). (E) Relationship between the intensity of afterglow signals and levels of normalized expression of CRT. (F) Correlation between the intensity of afterglow signals and the levels of ATP. (G) Schematic representation of ICD and its anticancer efficacy. (H) Afterglow images of 4T1 tumor‐bearing mice. (I) Correlation between the intensity of afterglow signals and expression level of CRT. (J) Relationship between signal intensities of afterglow observed post‐treatment and rates of tumor inhibition at 16 days. Reproduced with permission from ref. [93]. Copyright 2021, Wiley‐VCH.

Recently, Zhang et al. reported an organic afterglow nanoprobe (designated as PFODBT@CPPO) for the visualization of self‐generated ROS during PDT, enabling early prediction of therapeutic efficacy through near‐infrared afterglow luminescence [93]. In the nanoprobe system, PFODBT served as an initiator, substrate, and relay unit in the afterglow process, while CPPO acted as a magnifier of the afterglow. The thiophene group of PFODBT and CPPO were simultaneously targeted by ^1^O_2_, resulting in the formation of a thiophene‐dioxetane intermediate (Path I) and a dioxetanedione intermediate (Path II), respectively. Subsequently, these intermediates underwent cleavage due to unstable high‐energy excited state and excited adjacent PFODBT to emit afterglow via the intramolecular electron transfer mechanism. Furthermore, the excessive generation of ^1^O_2_ during this process can effectively re‐excite PFODBT@CPPO, thereby significantly amplifying the intensity of afterglow luminescence through a double‐cycle amplification pathway (Figure 6A).

In vitro experiments showed that afterglow intensity had a good relationship with the ^1^O_2_ generation and afterglow intensity was well correlated with cell inhibition rate (Figure 6B,C). Furthermore, ^1^O_2_ generated by PFODBT@CPPO triggered necrosis of cancer cell and boosted the release of damage‐based factors such as calreticulin (CRT) or adenosine triphosphate (ATP), thereby causing immunogenic cell death (ICD)‐related immune responses via ROS‐associated oxidative stress. In vitro experiments also demonstrated a significant correlation between the afterglow signals and the expression of ICD induced by the nanoprobe (Figure 6D–F). This suggested that the PFODBT@CPPO afterglow nanoprobe had potential for timely monitoring of both ^1^O_2_ yield and ^1^O_2_ mediated immune response during PDT.

Encouraged by afterglow luminescence imaging performance of PFODBT@CPPO, timely evaluation of therapy effect in vivo were carried out (Figure 6G). The injection dose was positively correlated with the afterglow signal intensity (Figure 6H), and the increased signal intensity observed in the tumor region after treatment suggested a higher level of CRT. (Figure 6I). In addition, the inhibition of tumor growth at 16 days post‐treatment exhibited a strong correlation with the intensity of tumor afterglow observed at 2 min post‐injection (Figure 6J), which exhibited that tumors with a low afterglow signal was slightly inhibited, while tumors with high afterglow signal showed significant shrink. The results presented herein demonstrate that this afterglow imaging strategy offers valuable insights for early prediction and timely evaluation of the therapeutic efficacy mediated by ROS in cancer treatment.

## 7 Early Assessment of Therapeutic Efficacy Using Multimodal Imaging Strategy

Multimodal imaging combines two or more imaging techniques to provide complementary anatomical and molecular information of a living subject, making it a valuable tool in basic biomedical research and clinical diagnosis [94, 95, 96, 97]. A single imaging technique frequently falls short in meeting the demands for both high sensitivity and specificity, effective targeting, precise spatial resolution, and exceptional imaging depth. The utilization of multimodal imaging allows for the integration of complementary benefits and significantly reduces the likelihood of missed diagnoses that may occur when relying solely on a single imaging modality to analyze or visualize physiological alterations [42, 98]. To date, reports of early evaluation of therapy effect using multimodal imaging are scarce.

**FIGURE 7:**
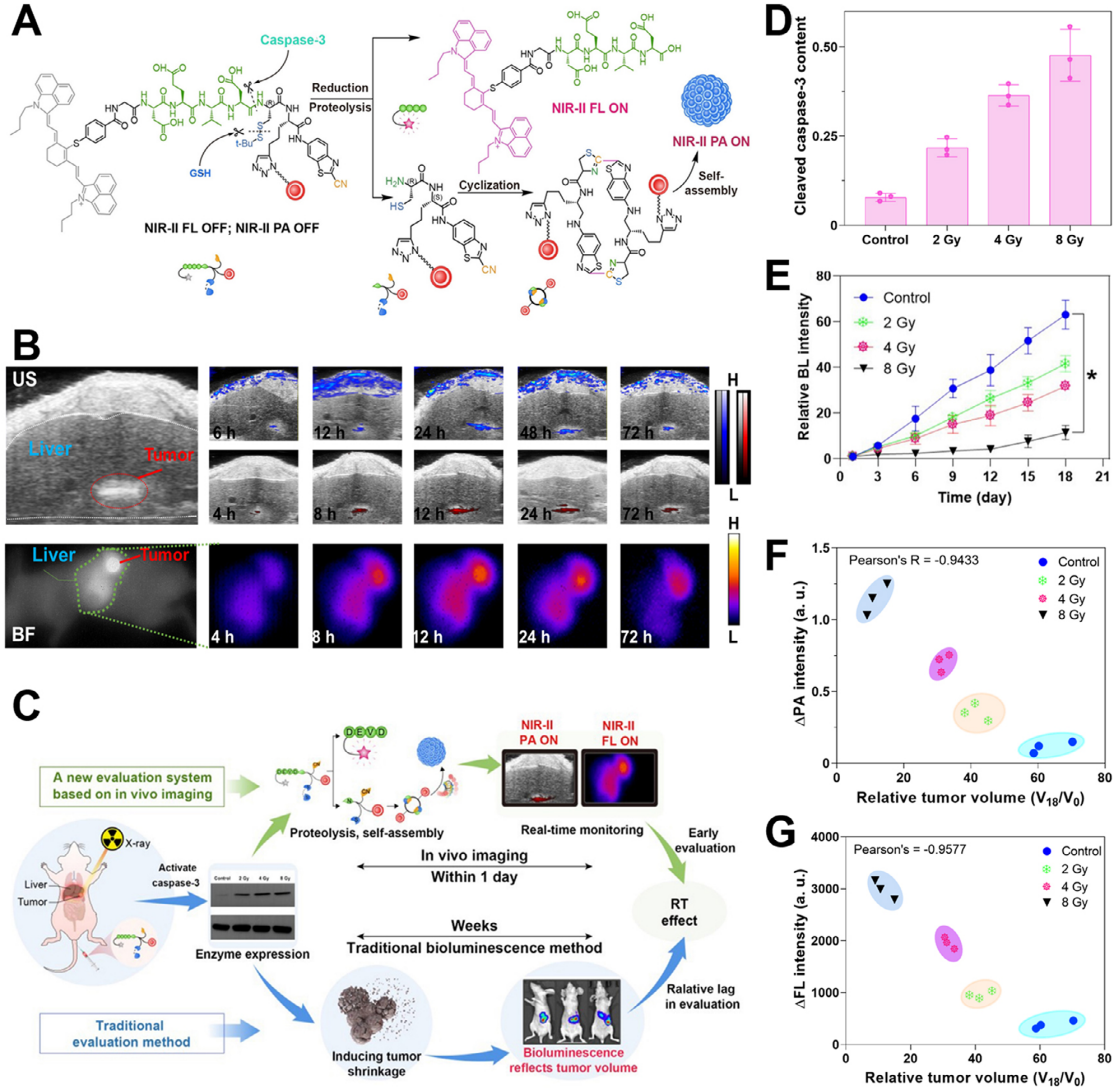
(A) Illustration depicting the activation of FL and photoacoustic (PA) signals through caspase‐3 enzyme‐induced enhancement. (B) Orthotopic liver cancer was imaged using PA at 680 nm (top) and 1250 nm (middle), as well as FL imaging at 1150 nm (bottom). (C) Schematic illustration of an early evaluation system. (D) Comparison of cleaved caspase‐3 levels following exposure to varying doses of X‐ray radiation. (E) The relative intensity of bioluminescence. (F) Correlation between ΔPA intensity at 12 h‐post RT, (G) ΔFL intensity at 12 h‐ and the relative tumor size at 18 days‐post RT, respectively. Reproduced with permission from ref. [99]. Copyright 2022, Wiley‐VCH.

Fu et al. developed a bimodal nanoprobe (referred to as AuNNP@DEVD‐IR1048) that utilizes photoacoustic (PA) and FL imaging techniques to detect caspase‐3 apoptosis enzyme activity. This nanoprobe enables early prediction and timely assessment of the effects of radiotherapy [99]. The nanosensor connected the nanogapped AuNPs (AuNNPs serving as energy acceptors) and NIR‐II FL dyes (IR‐1048 acting as energy donors) using a polypeptide substrate responsive specifically to caspase‐3 (DEVD). The nanoprobe exhibited a diminished PA signal and reduced FL signal within the NIR‐II range as a result of acceptors and donors undergoing FRET interaction. The amide bond between DEVD and cysteine (Cys) in the nanoprobe could be cleaved by the caspase‐3 enzyme, resulting in the release of IR‐1048 and activation of NIR‐II FL signal. This process is accompanied by Cys(StBu)‐(AuNNP)‐CBT, which undergoes a click condensation reaction with CBT‐Cys to facilitate self‐assembly into aggregates of AuNNPs. The plasmonic coupling effect between these adjacent AuNNPs leads to enhanced NIR‐II PA signal (Figure 7A).

After being administered intravenously, the nanoprobes exhibited accumulation at the tumor site. The maximum accumulation of nanoprobes within the tumor was observed 24 h after injection, as indicated by NIR‐I PA imaging. After exposure to X‐ray radiation at this specific time interval, the activation of caspase‐3 enzyme resulted in the cleavage of DEVD through proteolysis. This subsequently led to the simultaneous generation of both NIR‐II PA and FL imaging signals, with the peak signal intensity observed 12 h after irradiation (Figure 7B). Subsequently, NIR‐II PA and FL imaging were utilized for the in vivo early prediction and timely assessment of RT effect (Figure 7C). The correlation between irradiation doses and the expression levels of caspase‐3 was demonstrated by the results obtained from NIR‐II PA enhancements (ΔPA), NIR‐II FL intensity enhancements (ΔFL), and cleaved caspase‐3 expression at 12 h after irradiation (Figure 7D). This finding suggested the potential for developing a timely assessment system to evaluate therapy effectiveness, as activated caspase‐3 can activate both NIR‐II FL and PA signals, leading to tumor growth inhibition through caspase‐3‐related cell apoptosis pathways. The traditional bioluminescence imaging technique was used to monitor tumor size (Figure 7E), revealing a strong correlation between relative tumor size (*V*_18_/*V*_0_) and ΔPA intensity at 12 h post‐irradiation (Figure 7F) or ΔFL intensity (Figure 7G). As the RT activated caspase‐3 plays a crucial role in connecting imaging data with tumor growth rate, it is possible to establish a correlation mechanism between the intensity of molecular imaging signals and the effectiveness of RT. This could potentially offer an innovative approach for timely assessment and early prediction of treatment outcomes.

RT is a widely employed clinical strategy for cancer treatment, involving the irradiation of tumor tissues using ionizing beams such as X‐rays. Increasing evidence suggests that the generation of hydroxyl radicals (·OH) through radiolysis of water molecules plays a pivotal role in inducing apoptosis in cancer cells, as more than 50% of DNA damage observed in standard radiotherapy can be attributed to ·OH. Therefore, real‐time monitoring of ·OH production in tumors during RT may provide invaluable insights for early assessment of therapeutic efficacy and optimization of treatment strategies. Ye et al. developed an optimization strategy for a diene electrochromic material (1‐Br‐Et) as a highly responsive chromophore to ·OH, which has been ingeniously utilized to fabricate a cutting‐edge NIR ratiometric FL and PA bimodal probe for in vivo visual imaging of ·OH [100]. Remarkably, this innovative probe exhibited an extraordinary FL ratio ranging from 780 to 1113 nm, while maintaining a commendable PA ratio between 755 and 905 nm. By virtue of the oxidation process of 1‐Br‐Et by ·OH, the FL_780_/FL_1113_ decreases significantly, whereas the PA_755_/PA_905_ simultaneously increases substantially. Consequently, this innovative technology facilitates precise monitoring of ·OH production in tumors exposed to RT, enabling early prediction of therapeutic efficacy.

## 8 Conclusion and Prospects

The delay in assessing the effectiveness of treatment presents a considerable obstacle to improving the quality of life for individuals with unresponsive forms of cancer. Early stratification and timely evaluation of therapy effect are vital for optimizing therapeutic regime and prolonging the survival of patients. With the swift advancement of intelligent nanomaterials and molecular imaging, significant advancements have been achieved in diverse activatable nanoprobes and imaging methodologies. Strategies that combine activatable probes and molecular imaging techniques have shown great promise in the early stratification and timely evaluation of therapy effect with noninvasiveness, high sensitiveness, and accuracy. This review dived into the topic of using molecular imaging with biomarker activatable probes techniques to stratify and evaluate the effect of cancer treatment (Table 1). Compared to conventional method based on stratification and evaluation of therapy effect by measuring tumor size changes before and after treatment, molecular imaging with activatable nanoprobe is a timelier and more sensitive channel for assess the tumor therapeutic response, which is a great benefit for guiding doctors to timely evaluate therapy effect and formulate optimal therapeutic regime for patients.

**TABLE 1:** Biomarkers and their corresponding imaging probes, along with their application.

Biomarker	Corresponding imaging probe	Application	Ref.
Programmed cell death protein 1 (PD‐1)	αPDL1‐AuNPs	CT imaging‐mediated early stratification of therapeutic response to immunotherapy	[48]
Cell‐surface biomarker of T cell activation (OX40)	^64^Cu‐DOTA‐AbOX40	Early predict immunotherapy effect via PET	[55]
Immunoactivation‐related biomarkers (granzyme B)	^68^GaNOTA‐GZP	Early stratification of therapy response via PET	[58]
Dendritic cells (DCs)	IO‐RNA‐NPs	Early predict antitumor response with MR imaging	[66]
Reactive oxygen species (ROS)	IO‐Gd NVs	Early stratification of RT effect through activatable inflammation MR imaging	[67]
Hypoxia‐inducible factor	MR‐CA	Early stratification to predict both RT and immunotherapy effect through MR imaging	[68]
Granzyme B	CyGbPF and CyGbPP	Timely evaluation of immunotherapy effect through FL imaging	[79]
Acidic tumor microenvironment (pH)	LET‐1052	Early prediction and timely assessment of PTT effectiveness through FL imaging	[80]
pH	BiRN	Early evaluation of chemotherapy effect and early stratification of patients for personalized cancer therapy via ratiometric FL imaging	[81]
ROS	PFODBT@CPPO	Early evaluation of therapeutic efficacy through afterglow luminescent imaging	[93]
Apoptosis enzyme (caspase‐3)	AuNNP@DEVD‐IR1048	Timely assessment and early prediction of treatment outcomes through multimodal imaging	[99]
Hydroxyl radicals (·OH)	1‐Br‐Et	Precise monitoring of ·OH production in tumors exposed to RT, enabling early prediction of therapeutic efficacy.	[100]

While there exist appealing avenues for the advancement of early stratification and timely assessment of therapy impact using molecular imaging with biomarker responsive probes, it is recommended that future investigations concentrate on, without confining their inquiries to, the subsequent areas: (1) Seeking novel biomarkers that are more sensitively and accurately reflect the pathological environments, designing this biomarkers activatable probes to improve the timely, reliability, specificity of evaluation. (2) Developing cascaded multi‐biomarkers activatable probes that enables sequential multi‐stimuli induced imaging signal activation/amplification for providing accurate evaluation of therapy effect. (3) Setting up an activatable nanoprobes with multi‐modal imaging capability to realize the complementary advantages of multiple imaging modalities, which greatly lowers the probability of wrong judgment in evaluation of therapy effect. (4) Designing an activatable ratiometric nanoprobes to enable self‐calibrate therapy effect, which is also propitious to accurately evaluating the therapy effect in real time.

## Conflicts of Interest

The authors declare no conflicts of interest.
